# Long-Lasting Priming of Endothelial Cells by Plasma Melatonin Levels

**DOI:** 10.1371/journal.pone.0013958

**Published:** 2010-11-12

**Authors:** Eduardo Koji Tamura, Pedro Augusto Fernandes, Marina Marçola, Sanseray da Silveira Cruz-Machado, Regina Pekelmann Markus

**Affiliations:** Laboratory of Chronopharmacology, Department of Physiology, Institute of Bioscience, Universidade de São Paulo, São Paulo, São Paulo, Brazil; Maastricht University, Netherlands

## Abstract

**Background:**

Endothelial cells are of great interest for cell therapy and tissue engineering. Understanding the heterogeneity among cell lines originating from different sources and culture protocols may allow more standardized material to be obtained. In a recent paper, we showed that adrenalectomy interferes with the expression of membrane adhesion molecules on endothelial cells maintained in culture for 16 to 18 days. In addition, the pineal hormone, melatonin, reduces the adhesion of neutrophils to post-capillary veins in rats. Here, we evaluated whether the reactivity of cultured endothelial cells maintained for more than two weeks in culture is inversely correlated to plasma melatonin concentration.

**Methodology/Principal Findings:**

The nocturnal levels of melatonin were manipulated by treating rats with LPS. Nocturnal plasma melatonin, significantly reduced two hours after LPS treatment, returned to control levels after six hours. Endothelial cells obtained from animals that had lower nocturnal melatonin levels significantly express enhanced adhesion molecules and iNOS, and have more leukocytes adhered than cells from animals that had normal nocturnal levels of melatonin (naïve or injected with vehicle). Endothelial cells from animals sacrificed two hours after a simultaneous injection of LPS and melatonin present similar phenotype and function than those obtained from control animals. Analyzing together all the data, taking into account the plasma melatonin concentration versus the expression of adhesion molecules or iNOS we detected a significant inverse correlation.

**Conclusions/Significance:**

Our data strongly suggest that the plasma melatonin level primes endothelial cells “in vivo,” indicating that the state of the donor animal is translated to cells in culture and therefore, should be considered for establishing cell banks in ideal conditions.

## Introduction

Endothelial cells are located on the internal vascular layer and are responsible for modulating vascular tone and leukocyte migration. They are of great interest to cell therapy and tissue engineering. The heterogeneity among cells originated from different sources and culture protocols needs to be understood in order to obtain more standardized material to avoid rejection due to uncontrolled migration of immune competent cells [1].

Endothelial cells are known to be preconditioned “in vitro”. Treatment with low concentrations of lipopolysaccharide (LPS) or tumor necrosis factor (TNF) [2], and short periods of ischemia-reperfusion (I/R) [3] protect the cells against subsequent harmful injuries. LPS activates the nuclear factor kappa B (NFKB) pathway, which leads to the expression of inducible nitric oxide synthase (iNOS) [4]. Melatonin, the indoleamine derived from serotonin and released at night by the pineal gland, inhibits LPS-induced iNOS expression [4] and leukocyte-endothelial interaction [5], [6]. The short and long-term effects of melatonin are due to modulation of calcium metabolism [7], and inhibition of the NFKB pathway [4], respectively.

During the last decades, many works showed the immune properties of melatonin, and more recently, we suggested the existence of an immune-pineal axis [8]. In such context, LPS and the inflammatory cytokine TNF, produced at the beginning of an inflammatory response, inhibits pineal gland nocturnal melatonin production [9], [10], which is restored by corticosterone [11]. Taking into account that melatonin inhibits the rolling and adhesion of leukocytes to endothelial cells [5], the reduction in plasma melatonin levels favors the migration of cells to the site of a lesion. A similar role is exerted by glucocorticoids, as adrenalectomy or inhibition of corticosterone synthesis also result in an increase in leukocyte rolling and adhesion to post-capillary veins [12].

Cultures of endothelial cells obtained from adrenalectomized rats adhere significantly more neutrophils than those obtained from sham operated or naïve rats [13], indicating that these cells could be primed by “in vivo” conditions. Taking into account that a lack of adrenal glands is not a common condition, we looked for other models that could prime endothelial cells.

The present work aimed to evaluate whether plasma melatonin level could interfere in the reactivity of endothelial cells cultured up to 16–18 days. The pineal gland is an integral part of the innate immune response, as the production of melatonin is suppressed in order to allow a full migration of neutrophils independently of the hour of the day [8]. Thus, we injected LPS in order to reduce nocturnal melatonin surge. As a negative control, rats were injected with LPS plus melatonin. We measured the adhesion of leukocytes to primed endothelial cells, as well as the expression of molecules related to endothelium activation, such as adhesion molecules and iNOS. The inverse correlation between plasma melatonin level and the reactivity of endothelial cells strongly suggest a priming of the cells by the status of the donor at the moment of death.

## Materials and Methods

**Figure 1:**
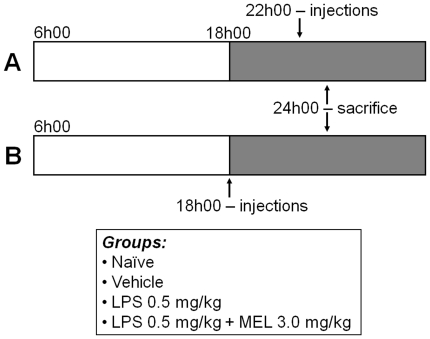
Experimental protocols. Wistar rats were divided into four groups: naïve, vehicle, lipopolysaccharide (LPS, 0.5 mg/kg) and LPS + melatonin (MEL 3 mg/kg). The intravenous (caudal vein) injections of vehicle, LPS or LPS + melatonin were performed two (A) or six (B) hours before sacrifice (6 hours after lights off –24h00). Grey portion represents the dark phase of the day.

Adult Wistar rats (300–330 g), housed under 12/12 h light/dark cycle (lights on at 06h00), were divided into four groups: naïve, vehicle (saline +5% ethanol), LPS (0.5 mg/kg; Sigma) and LPS + melatonin (3 mg/kg; Sigma). All rats were killed six hours after lights off, which represent the maximal plasma melatonin concentration [11]. LPS injection (intravenous – caudal vein) was done two or six hours before decapitation (Fig. 1), in order to coincide with the TNF plasma peak (two hours) and with the return to basal levels (six hours) [14].

All experiments were carried out in compliance with ethical standards of our institution (Ethic Committee of the Institute of Bioscience of the University of São Paulo license 086/2008) and with the recommendations of the National Council on Experimental Animal Control (CONCEA).

### Melatonin assay

Plasma melatonin was assayed using an ELISA kit with a detection limit of 3.0 pg/mL (GenWay Biotech Inc., CA, USA). The plates were read in a microplate spectrophotometer (SpectraMAX 250, Molecular Devices, CA, USA) at 405 nm.

### Endothelial cell culture

Primary cultures of endothelial cells were obtained from rat cremaster muscle according to the method described previously [15]. The cremaster was isolated, washed with phosphate saline solution (PSS; mM: NaCl 125.0, Na_2_HPO_4_ 2.0, NaH_2_PO_4_ 2.0 and KCl 5.0) and cut into pieces of approximately 2×2 mm. Two pieces per well were placed into a 24-well culture plate with Dulbecco's Modified Eagle Medium (DMEM, Gibco) plus gentamicin (40 mg/L, Gibco) and fetal bovine serum (20%, Gibco). The explants were maintained in a humidified incubator (37°C, 5% CO_2_). After 48 h the explants were removed, the cells were cultured till confluence (14–16 days), and the medium was changed every 48 h.

### Immunofluorescence

Protein detection by immunofluorescence was based on Tamura et al. [4]. Endothelial cells were subcultured for 48 h in chamber slides (8 wells, 10^4^ cells/well), fixed in methanol/acetone (1∶1, 15 min, −20°C) and incubated with antibodies for ICAM-1 or PECAM-1 (1∶100) for 20 min (FITC-conjugated mouse anti-rat ICAM-1; intercellular adhesion molecule 1; PE-conjugated mouse anti-rat PECAM-1; platelet endothelial cell adhesion molecule 1; BD Pharmingen), or permeabilized for 30 min (room temperature) with Triton X-100 (0.2%) and incubated overnight (4°C) with iNOS antibody (1∶50, TRITC-conjugated polyclonal rabbit anti-iNOS Santa Cruz Biotechnology). FITC and PE were excited at 488 nm (Argon laser) and 543 nm (HeNe laser) and emitted fluorescence was measured at 515–530 and 560–575 nm, respectively. TRITC was excited at 543 nm and emitted fluorescence at 514 nm. Slides mounted in glycerol/PSS solution (1∶1) were visualized by confocal microscopy (LSM 510; Carl Zeiss, Jena, Germany). Three fields with 4–6 cells were randomly chosen and imaged.

### Leukocyte-endothelial cell adhesion assay

The adhesion assay was performed according to Lotufo et al. [6]. Endothelial cells (10^4^ cells/well) from different groups were seeded in 96-well plates and allowed to grow till confluence, which was reached after 5 days.

For each experimental group, one well of endothelial cells was kept without polymorphonuclear cells (PMN). At the end of the experimental protocol we counted the number of endothelial cells in these wells, which was used to normalize the amount of PMN adhered. This procedure was important, because the amount of endothelial cells after reaching confluence could be different in each experimental group.

**Figure 2:**
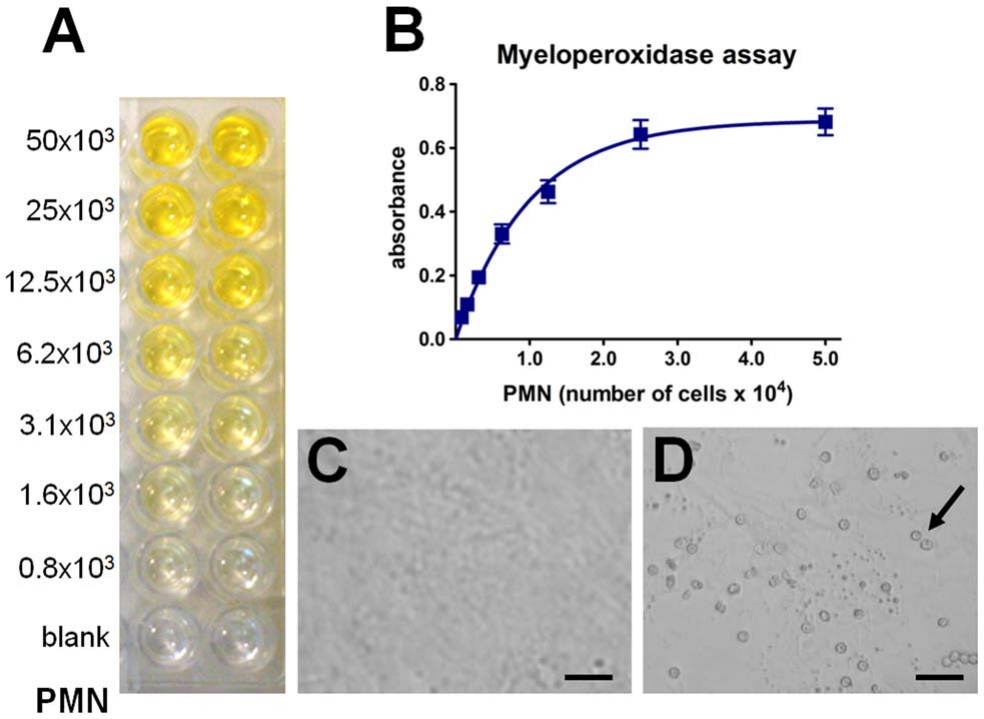
Standard curve of leukocyte adhesion assay. Representative image of experimental myeloperoxidase assay (A). The number of polymorphonuclear cells (PMN) was determined by comparison with standard curve (B). Confluent endothelial cell culture adhered (D) or not (C) with leukocytes. Arrow indicates leukocytes adhered to endothelial cells. Scale bar  = 50 µm.

At the day of the experiment, endothelial cells, washed with Hank's balanced salt solution, were incubated for 30 min with PMN (5×10^4^ cells/well) obtained from rat aorta blood. Nonadherent PMN were washed and myeloperoxidase colorimetric assay was applied to detect the PMN adhered to the endothelial cells layer, using tetrametylbenzidine (TMB; Sigma) as the peroxidase substrate. Cells were incubated at room temperature for 5 min with 0.25% of dodecyltrimethylammonium bromide as a peroxidase solubilizer and TMB (5,3 µM) dissolved in sodium acetate buffer (0.05 M, pH 5.8). In sequence, hydrogen peroxidase (100 µL, 2.5 mM) was incubated for exactly 2 min, and the reaction was stopped with sulfuric acid (50 µL, 1 M). The absorbance was determined using a microplate spectrophotometer (spectraMAX 250, Molecular Devices, CA, USA) at 420 nm. The quantification of PMN adhesion was based on a standard curve obtained with known amounts of PMN. The number of adhered cells was calculated from the one-phase association equation obtained from the standard curve using Graphpad Prism Software (4.0; CA, USA) (Fig. 2).

### Statistical analysis

All the data are expressed as mean ± S.E.M., and were compared by Student's *t*-test (two groups) or one-way ANOVA followed by Newman-Keuls test (more than two groups). Linear regression was tested by Analysis of Variance for detecting slopes (expressed as mean ± S.E.M.) different from zero, and correlation between two parameters was tested according to Pearson's coefficient. Results were considered significantly different when P<0.05. Plasma melatonin is shown as pg/mL and the other results as percentage of naïve group, set as 100%.

## Results

### Plasma melatonin

**Figure 3:**
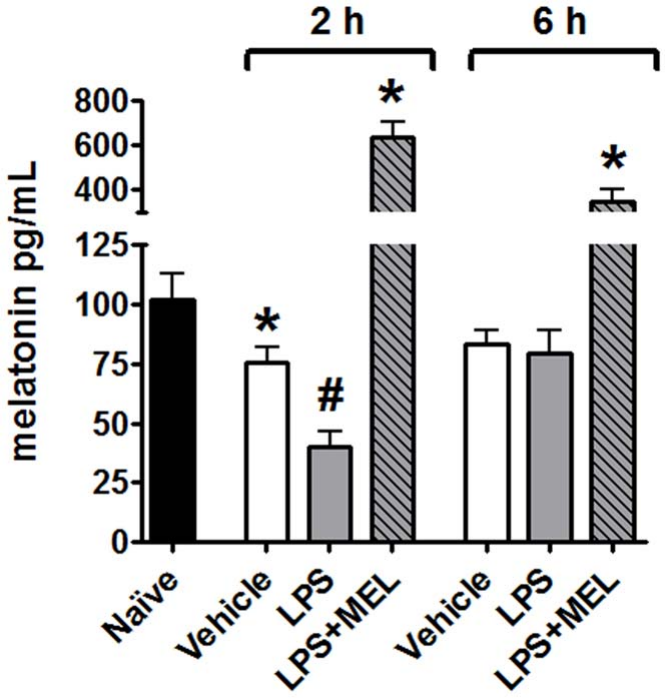
Nocturnal plasma melatonin levels vary during the development of response against lipopolysaccharide challenge. Plasma melatonin from naïve (black bar), vehicle injected (white bars), lipopolysaccharide injected (LPS, 0.5 mg/kg; grey bars) or LPS plus melatonin injected (3 mg/kg; grey diagonal striped bars) animals was determined by ELISA. The injections were performed 2 or 6 hours before sacrifice (6 hours after lights off –24 h 00). Data are expressed as mean ± SEM, n = 4–8 per group of at least two different experiments; * significantly different (P<0.05) versus naïve group (black bar); # significantly different (P<0.05) versus the 2 hours vehicle group (white bars).

Plasma melatonin concentration was significantly reduced after two, but not after six hours of LPS injection when compared to the vehicle (Fig. 3). A smaller, but significant, reduction in melatonin was also observed when the group injected with vehicle (2 hours) was compared to naïve group. Here, there was no difference after six hours. The administration of 3 mg/kg of melatonin increased the plasma concentration of melatonin to values much higher than those determined at night.

### Leukocytes adhesion

**Figure 4:**
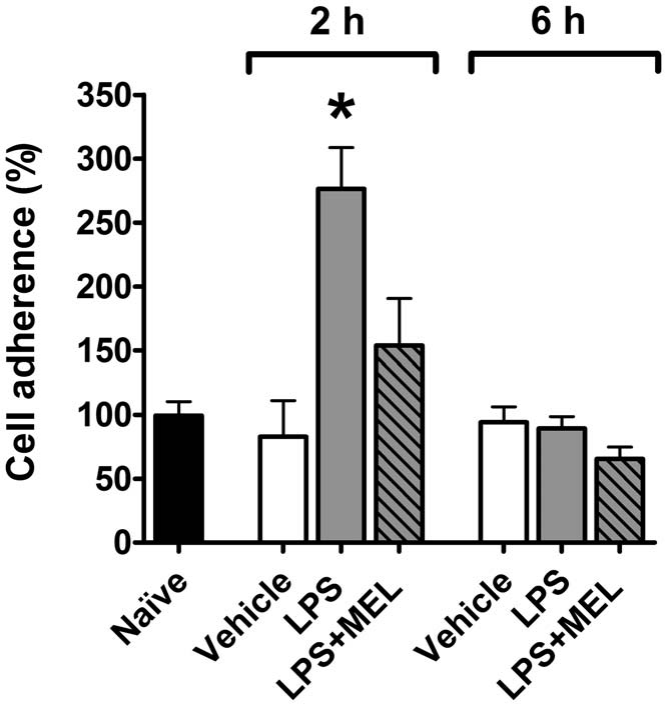
Melatonin regulates leukocyte adhesion to cultured endothelial cells. Leukocyte adhesion on endothelial cells cultures obtained from naïve (black bar), vehicle injected (white bars), lipopolysaccharide injected (LPS, 0.5 mg/kg; grey bars) or LPS + melatonin injected (MEL 3.0 mg/kg; grey diagonal striped bars) animals was determined by leukocyte-endothelial adhesion assay. The injections were performed 2 or 6 hours before sacrifice (6 hours after lights off –24 h 00). Data are expressed as mean ± SEM, n = 4–8 per group of at least two different experiments; * significantly different (P<0.05) versus 2 hours vehicle group (white bars).

LPS injected two hours before sacrifice increased the adhesion of leukocytes to endothelial cells in comparison to naïve and vehicle groups. Conversely, six hours after LPS, no difference between adherence in experimental and control groups were observed. No difference between naïve and vehicle groups was observed. Melatonin treatment inhibited the effect of LPS in the group sacrificed two hours after treatment (Fig. 4).

### iNOS expression

**Figure 5:**
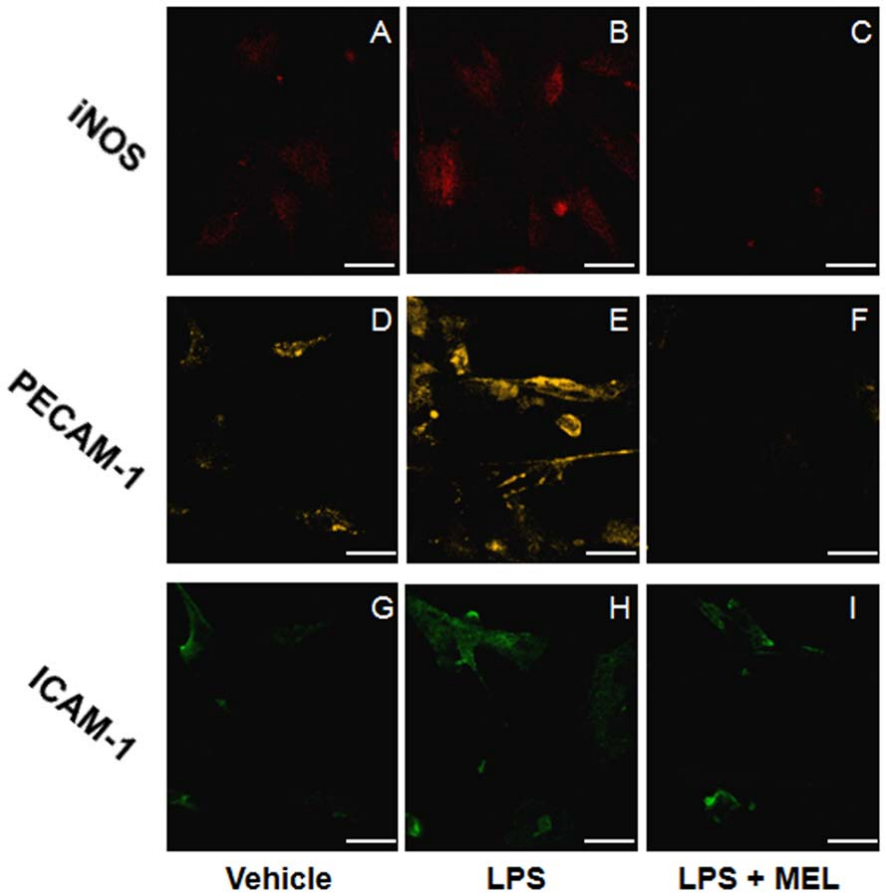
Melatonin regulates the lipopolysaccharide-induced iNOS, PECAM-1 and ICAM-1 expression in endothelial cells. Representative images of immunofluorescence assays of iNOS (A–C), PECAM-1 (D–F) and ICAM-1 (G–I). Columns show cells obtained from vehicle (A, D and G), lipopolysaccharide (LPS) (B, E and H) and LPS + melatonin (MEL) (C, F and I) injected animals. Scale bar  = 50 µm.

**Figure 6:**
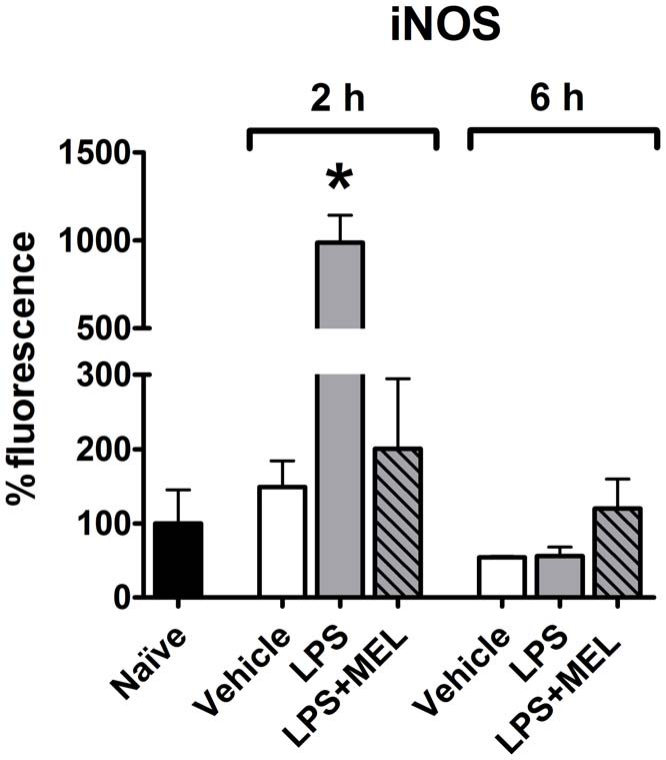
Melatonin regulates the lipopolysaccharide-induced iNOS expression in endothelial cells. iNOS expression in cultures obtained from naïve (black bar), vehicle injected (white bars), lipopolysaccharide injected (LPS, 0.5 mg/kg; grey bars) or LPS + melatonin injected (MEL 3.0 mg/kg; grey diagonal striped bars) animals was determined by confocal immunofluorescence. The injections were performed 2 or 6 hours before sacrifice (6 hours after lights off –24h00). Data are expressed as mean ± SEM, n = 4–8 per group; *significantly different (P<0.05) versus 2 hours vehicle group (white bars).

iNOS expression in endothelial cells from the LPS group injected two hours before sacrifice was increased in comparison to naïve and vehicle groups. On the other hand, six hours after LPS, no difference was observed. Melatonin inhibited the effect of LPS in the group sacrificed two hours after treatment (Fig. 5 A, B, C; figure 6).

### PECAM-1 and ICAM-1 expression

**Figure 7:**
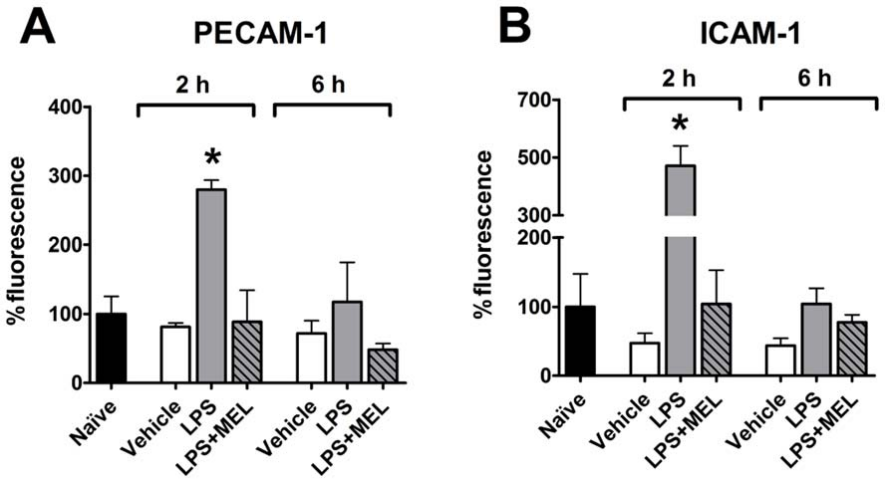
Melatonin regulates the lipopolysaccharide-induced PECAM-1 and ICAM-1 expression in endothelial cells. Expression of PECAM-1 (A) and ICAM-1 (B) in cultures obtained from naïve (black bar), vehicle injected (white bars), lipopolysaccharide injected (LPS, 0.5 mg/kg; grey bars) or LPS + melatonin injected (MEL 3.0 mg/kg; grey diagonal striped bars) animals were determined by confocal immunofluorescence. The injections were performed 2 or 6 hours before sacrifice (6 hours after lights off –24h00). Data are expressed as mean ± SEM, n = 4–8 per group; * significantly different (P<0.05) versus 2 hours vehicle group (white bars).

LPS administration two, but not six, hours before sacrifice induced an increase in PECAM-1 (Fig. 5D, E and 7A) and ICAM-1 (Fig. 5G, H and 7B) expression when compared to control groups. Melatonin impaired LPS-induced increase in the expression of these adhesion molecules (Fig. 5F and 5I). As occurred for the adhesion of leukocytes and iNOS expression, no difference in PECAM-1 and ICAM-1 was observed six hours after LPS injection when compared to control groups (Figs. 7A and 7B, respectively).

### Correlation between plasma melatonin and the expression of adhesion molecules and iNOS

**Figure 8:**
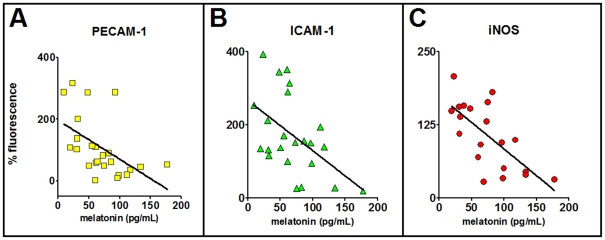
Plasma melatonin concentration is inversely correlated with the expression of adhesion molecules and iNOS in endothelial cells. The plasma concentration of melatonin (pg/mL) in each rat was plotted against the expression of PECAM-1 (A), ICAM-1 (B) and iNOS (C) shown as the percentage of the fluorescence in the naïve group seeded in the same chamber slide. The values of the slopes and the Pearson's coefficient are shown in the text.

The distribution of the data obtained by correlating the plasma concentration of melatonin versus the expression of adhesion molecules or iNOS, for each rat, followed linear regressions that were significantly different from zero when tested by analysis of variance for linear regression (Fig 8). The slopes of the lines for PECAM-1, ICAM-1 and iNOS were −1.13±0.48, −1.25±0.44 and −0.91±0.22, respectively. In addition, the Pearson's coefficient also point to an inverse correlation between the plasma concentration of melatonin and the expression of adhesion molecules (PECAM-1 and ICAM-1) or iNOS (Pearson's r = −0.189, −0.477 and −0.692; confidence limits −0.763 to −0.146; −0.754 to −0.057 and −0.869 to −0.360; P<0.009, 0.029 and 0.0007).

## Discussion

The increasing interest in applying cell therapy needs to be preceded by improving the process of obtaining homogeneous and reliable cell cultures. Endothelial cells, which form an interface between blood and vascular tissue, regulate vascular tonus and immune response. These cells can be obtained in a quiescent or activated state, depending on the health of the donor. In the activated state, cultured endothelial cells should have more leukocytes adhere to them than quiescent cells, and their use in vascular grafts or tissue engineering could be harmful for the recipient. We have previously shown that melatonin reduces rolling and adhesion of leukocytes to the post-capillary vein endothelial layer [5]. In the present paper, we evaluated whether melatonin could have a protective effect against LPS-induced priming of endothelial cells.

Here, for the first time, we demonstrate that endothelial cell cultures obtained from LPS-treated animals have a higher ability to adhere leukocytes than cultures obtained from naïve or vehicle treated animals. This effect is strictly related to the time-course of the innate immune response, as only cells derived from animals killed two, but not six, hours after LPS were in an activated state, since they exhibit a higher expression of adhesion molecules and iNOS when compared to controls. It is interesting to note that the “in vitro” addition of LPS has a similar, but slower time-course than that observed after “in vivo” priming. The incubation of human umbilical vein endothelial cells (HUVEC) with LPS leads to a maximal increase in leukocytes adhesion in four to six hours, and even after 24 hours the cells are still activated [16].

Melatonin concentration in the plasma of donor animals was reduced after two, but not six hours after LPS treatment, strongly suggesting its relevance in the priming of endothelial cells. The plasma TNF maximal peak in rats is supposed to occur two hours after LPS treatment, while after six hours the level of TNF returns to basal values [14]. The time-course of plasma melatonin variation shown here reinforce a putative inhibition of pineal melatonin synthesis by TNF. We have recently shown that LPS inhibits nocturnal melatonin production by activating toll-like receptor 4 (TLR4) expressed in the pineal gland, and leading to a local production of TNF [9]. The activation of LPS and TNF receptors is transcribed by NFKB pathway [9]. This effect implies in the reduction in nocturnal melatonin surge. Activation of NFKB in the pineal gland inhibits the transcription of the key enzyme in melatonin synthesis, arylalkylamine-*N*-acetyltransferase [10], [17]. Thus, LPS blocks nocturnal melatonin surge.

In order to confirm the role of melatonin in priming endothelial cells, we tested the response of cells obtained from animals treated with LPS plus melatonin. In this case, the state of the endothelial cells was similar to that of the control, suggesting that the reduction of melatonin by LPS was responsible for increasing endothelial cell activity. However, the concentration of melatonin attained in the plasma of animals injected with LPS plus melatonin was much higher than that observed in the vehicle group.

In order to verify whether plasma melatonin concentration is in fact the endogenous factor that translates LPS effect, we analyzed the correlation between plasma melatonin concentration and the expression of adhesion molecules and iNOS for each animal. These results clearly show a significant inverse correlation, strongly suggesting that melatonin primes endothelial cells regarding the expression of adhesion molecules and iNOS.

The mechanism of action of melatonin could be related to the inhibition of the NFKB pathway, which was shown in immune competent cells [18], pineal gland [19] and in endothelial cells [4]. This pathway is known to result in epigenetic effects that could be inherited independently from changes in the genome [20], which would therefore, be maintained in long-term cultured cells.

In summary, this study evaluated the effect of melatonin in priming endothelial cells. The LPS-induced reduction in plasma melatonin levels is reflected in cultures that express higher amounts of adhesion molecules and iNOS, and have more leukocytes adhered than control cells. The restoration of plasma melatonin concentration six hours after LPS, or at the simultaneous injection of LPS and melatonin (in order to avoid the initial plasma reduction) leads to cultures with the same phenotype as the controls. Therefore, the state of the donor animal primes the cells and should be considered for establishing cell banks in ideal conditions.
